# The Role of Shunt Occlusion During Extracorporeal Life Support

**DOI:** 10.21470/1678-9741-2022-0047

**Published:** 2023

**Authors:** Sudesh Prabhu, Siddhant Mehra, Ganesh Sambandamoorthy, Balasubramanian Shanmugasundaram, Rajesh G Hegde, Riyan Shetty, Tom R Karl

**Affiliations:** 1 Pediatric Cardiac Services, Narayana Institute of Cardiac Sciences, Bengaluru, India; 2 Queensland Paediatric Cardiac Research, Brisbane, Australia

**Keywords:** Cardiac Output, Low, Cardiopulmonary Resuscitation, Extracorporeal, Membrane Oxygenation, Heart Arrest, Heart Defects, Congenital, Cardiac Arrest

## Abstract

**Introduction:**

The current recommendation for systemic to pulmonary artery shunt (SPS) patients requiring extracorporeal life support (ECLS) is to keep the shunt open, maintaining a higher pump flow. The practice in our center is to totally occlude the shunt while on ECLS, and we are presenting the outcome of this strategy.

**Methods:**

This is a retrospective analysis of patients who underwent SPS for cyanotic congenital heart disease with decreased pulmonary blood flow and required postoperative ECLS between January 2016 and December 2020. ECLS indication was excessive pulmonary blood flow, leading to either refractory low cardiac output syndrome (LCOS) or cardiac arrest. All patients had their shunts totally occluded soon after ECLS establishment.

**Results:**

Of the 27 SPS patients who needed postoperative ECLS (13 refractory LCOS, 14 extracorporeal cardiopulmonary resuscitation), wherein the strategy of occluding the shunt on ECLS initiation was followed, 16 (59.3 %) survived ECLS weaning and eight (29.6%) survived to discharge.

**Conclusion:**

Increased flow to maintain systemic circulation for a SPS patient while on ECLS is an accepted strategy, but it should not be applied universally. A large subset of SPS patients, who require ECLS either due to cardiac arrest or refractory LCOS due to excessive pulmonary flow, might benefit from complete occlusion of the shunt soon after commencement of ECLS, especially in cases with frank pulmonary edema or haemorrhage in the pre-ECLS period. A prospective randomized trial could be ethically justified for the subset of patients receiving ECLS for the indication of excessive pulmonary blood flow.

## INTRODUCTION

Systemic to pulmonary artery shunt (SPS) is a high-risk procedure, but mortality has declined over the years, even though the procedure has been used increasingly to palliate patients with single-ventricle physiology^[1]^. When performed for tetralogy of Fallot with or without pulmonary atresia, SPS has had an operative mortality of 4% (with a higher risk of hospital death in neonates)^[2]^. When extracorporeal membrane oxygenation (ECMO) is instituted in a patient with SPS, a large proportion of the ECMO blood volume will flow into the pulmonary circulation, as the pulmonary vascular bed has lower resistance. This in turn may lead to underperfusion of the systemic circulation and result in systemic organ dysfunction.

Petrucci et al.^[3]^ reported that 3.1% of neonates undergoing SPS need postoperative extracorporeal life support (ECLS). This series from the Society of Thoracic Surgeons and the Congenital Heart Surgeons’ Society database included 1,273 neonatal SPS performed between 2002 and 2009, with a mortality of 7.2%. The mortality for SPS patients needing ECLS was 62%.

In a patient with SPS, indications for ECLS include overflow-related refractory low cardiac output syndrome (LCOS), cardiac arrest, partial or complete shunt thrombosis, ventricular dysfunction due to a cardiac surgical procedure, and/or prolonged myocardial ischemic time.

In most cases, when patients with SPS are placed on an extracorporeal circuit, they are managed without occluding the shunt, maintaining an ECLS flow at 150-200 ml/kg/min. Higher flows are sometimes employed in such cases to maintain adequate systemic and pulmonary perfusion in the parallel circulation. Another strategy to manage ECLS in such patients is to constrict the shunt. The Extracorporeal Life Support Organization (ELSO) Red Book suggests that a SPS should be left open during ECLS, as previous attempts to manage the shunt by totally occluding it have resulted in 100% mortality^[4]^. This is primarily based on a publication by Jaggers et al.^[5]^, which analysed their outcome in nine such patients, wherein all the patients in whom the shunt was occluded succumbed.

The ELSO Guidelines For Pediatric Cardiac Failure 2021 mention that some patients with SPS needing ECLS require SPS constriction to achieve sufficient systemic perfusion on ECMO^[6]^. In such patients, adjustment or removal of the partial occlusion device to balance pulmonary and systemic blood flow during weaning and a trial off ECMO will be required. The guidelines also mention that in stage 1 palliative surgery with SPS, higher ECMO flow may be required (150-200 ml/kg/min). Temporary shunt constriction to limit pulmonary blood flow and promote systemic blood flow may be necessary^[6]^.

We are presenting a retrospective analysis of the outcomes of our SPS patients needing postoperative ECLS. All of them needed ECLS because of excessive pulmonary blood flow leading to either refractory LCOS or cardiac arrest. They all had their shunts clip occluded soon after establishment of ECLS. Our data suggests that good outcomes can be achieved by totally occluding the shunt while on ECLS. A higher overall mortality in our data set can possibly be attributed to sepsis, which remains a serious problem in India.

## METHODS

### Ethics Clearance

The ethics committee of Narayana Institute of Cardiac Sciences approved the study (NHH/AEC-CL-2021-688) and waived the need for individual consent.

All patients who underwent SPS for cyanotic congenital heart disease with decreased pulmonary blood flow (univentricular/biventricular) and who required postoperative ECLS between January 2016 and December 2020 in our hospital were included in this retrospective analysis. All of them were considered unsuitable for total correction (additional comorbidities, severely hypoplastic branch pulmonary arteries [PAs], multiple ventricular septal defects, need for right ventricular to pulmonary arterial conduit in small babies, severe form of trisomy 21, etc) or received their first stage of single-ventricle palliation.

The procedures were performed under general anaesthesia with endotracheal intubation and heparin (200 units/kg for off-pump and 400 units/kg for on-pump procedure). Shunts were selected based on the body weight^[7]^. Cardiopulmonary bypass (CPB) support was used whenever there was need for an additional procedure (PA plasty, septectomy, etc) or when there was hemodynamic compromise.

After the procedure, patients were ventilated until stable in the intensive care unit (ICU). Systemic heparinization used during the procedure was not reversed, and intravenous heparin infusion was started in the ICU once the activated clotting time (ACT) fell < 180 seconds. Heparin was titrated to maintain ACT in the range of 150 to 180 seconds. Subsequently, oral aspirin was started at 5 mg/kg/day (maximum of 75 mg/day).

Postoperatively, patients were monitored for signs of an overflowing shunt (widening of pulse pressure, progressive decline in lung compliance, worsening serial chest X-rays) and signs of LCOS (tachycardia, increased core to peripheral temperature difference, decreased urine output, lactic acidosis, and increase in the arteriovenous oxygen difference). Patients with shunt overflow were ventilated with lower percentage of oxygen, higher positive end-expiratory pressure, and permissive hypercapnoea. In addition, inotropes were appropriated for LCOS and vasoconstrictors for improving diastolic coronary perfusion.

ECLS was instituted either for refractory LCOS or as a part of extracorporeal cardiopulmonary resuscitation (ECPR). All ECLS procedures were performed in the ICU. All patients had central venoarterial ECMO. The cannulae and ECMO circuit were selected as per the hospital protocol, which was adapted from the Seattle Children’s Hospital protocol (based on patient’s weight) (https://www.pedsurglibrary.com/apsa/view/Pediatric-Surgery-NaT/829154/all/Cannulation_for_ECLS). After the establishment of stable ECLS, baseline ventilation was started, and the shunt was occluded completely in all patients using a clip or a small vascular clamp. Inotropes were stopped when the child was on full ECLS and restarted when weaning was attempted. Once the hemodynamic and metabolic parameters had improved, patients were weaned from ECLS support, and ventilation was appropriated. The clip was then removed to re-establish pulmonary circulation. Stable patients were separated from ECLS, and the chest was subsequently closed. Ventilation and peritoneal dialysis (PD) were continued as per the need.

The indications for ECLS, duration of ECLS, ventilation duration, renal function and PD requirement, blood and blood product requirement, ECLS complications, successful wean from ECLS (survival after discontinuation of ECMO without the need for reinitiation of mechanical support for the next 48 hours), and survival to discharge were assessed^[8]^. Inotrope requirement (vasoactive inotropic score [VIS]) prior to ECMO initiation was also discerned^[9]^.

Stable patients were discharged on oral diuretics and antiplatelet agents after satisfactory chest X-ray and echocardiographic findings.

Procedural mortality was defined as per The Society of Thoracic Surgeons Database definition^[10]^.

### Statistical Analysis

The statistical analyses were conducted using IBM Corp. Released 2015, IBM SPSS Statistics for Windows, version 23.0, Armonk, NY: IBM Corp. Continuous variables were described as mean and mean ± standard deviation and were analysed using Student’s t-test. Medians with interquartile range (IQR) were used for variables with skewed distributions and were analysed using Mann-Whitney U test. Categorical variables were described by taking frequencies and percentages and were analysed using Chi-square test or Fisher’s exact test when appropriate.

## RESULTS

Four hundred fifty-eight SPS were performed during the study period (with a mortality of 9.4%), with 27 patients requiring ECLS in the postoperative period. There were 13 (48.1%) males and 14 (51.9%) females. There were two (7.4 %) neonates, 15 (55.6 %) infants, and 10 (37 %) children older than one year. Mean age at surgery was 12.7 months, and mean weight was 6.2 kg. There were 21 (77.8 %) biventricular hearts and six (22.2 %) univentricular hearts. Ten (37%) patients had antegrade flow and 17 (63%) had pulmonary atresia. Two (7.4 %) were on preoperative mechanical ventilation. Twenty-five (92.6 %) were elective and two (7.4 %) were emergency procedures. Eighteen (66.7 %) patients required additional procedures (six - PA plasty, eight - atrial septectomy, three - unifocalization, one - total anomalous pulmonary venous connection [TAPVC] rerouting). Twenty-two (81.5 %) patients had procedures on CPB (18 - additional procedures, four - hemodynamic instability). Median CPB duration was 64 minutes (IQR 45 to 99). Twelve patients needed cardioplegic arrest (eight - atrial septectomy, one - TAPVC rerouting, three - PA plasty), and median cross-clamping duration was 22 minutes (IQR 9.5 to 49).

Indication for ECLS was refractory LCOS in 13 (48.1 %) patients and ECPR in 14 (51.9 %) patients. ECLS was instituted for excessive pulmonary blood flow leading to refractory LCOS or cardiac arrest. There was no requirement of ECLS for blocked shunt during the study period. ECMO was instituted via the trans-sternal approach with aortic and right atrial cannulation in all patients. One patient needed an additional inferior vena cava cannulation. Median time to establish ECPR ECMO was 32.9 minutes (range 20 - 45 minutes). The left heart was vented in one (3.7 %) patient (left atrial cannula), but additional 12 patients had an unrestrictive interatrial communication leading to left heart decompression while on ECLS (eight - post septectomy, one - single-ventricle TAPVC, three - unrestrictive atrial septal defect).

Mean time interval between surgery and ECMO initiation was 16.9 hours. Twenty-three (85.2 %) patients required ECLS while on mechanical ventilator support and four (14.8 %) after reintubation for hemodynamic compromise.

**Fig. 1:**
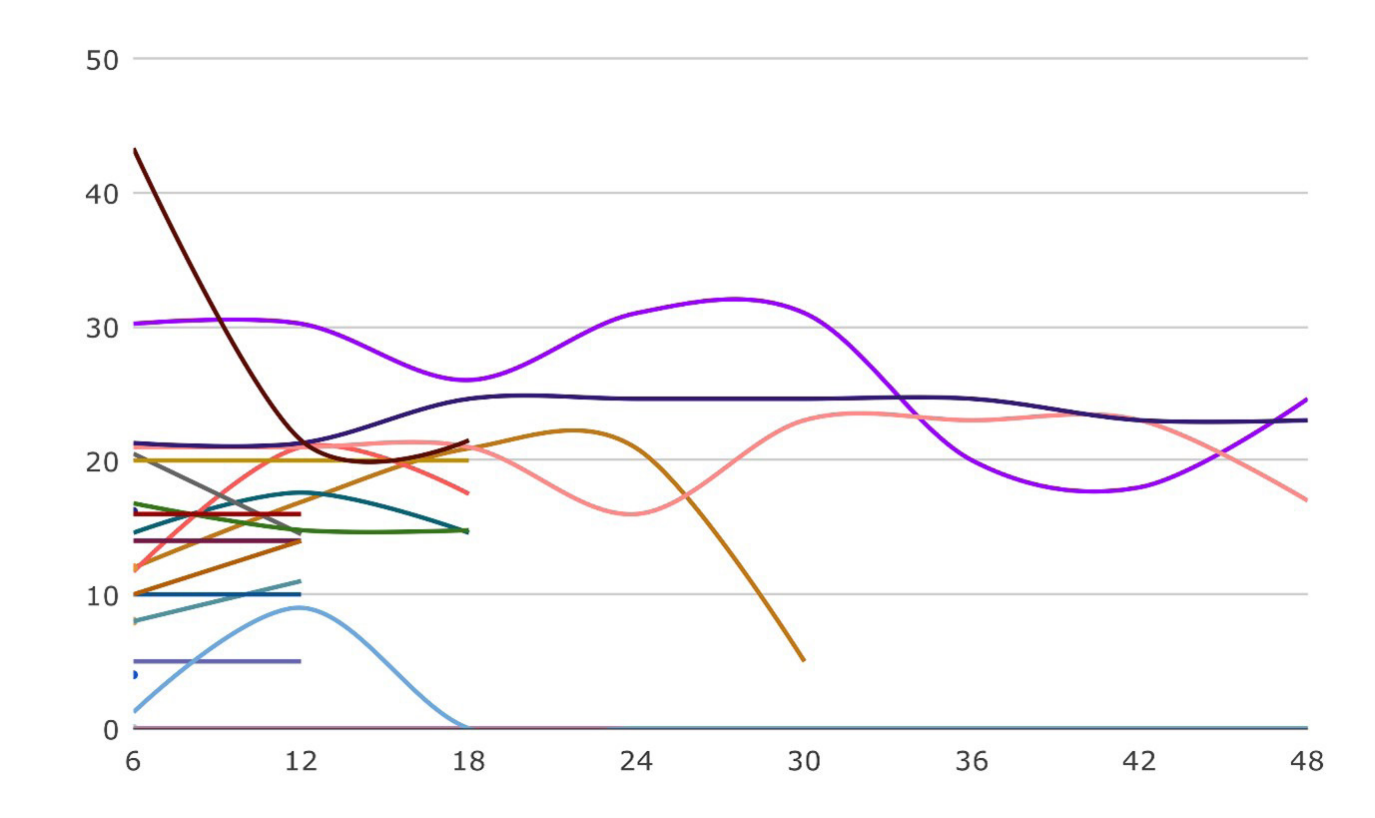
Vasoactive inotropic score (VIS) at six-hour intervals from surgery until initiation of extracorporeal life support (ECLS). In four patients, ECLS was initiated within six hours from surgery, hence it could not contribute for VIS trend.

In our series, VIS score was trending downwards (Figure 1) prior to ECMO initiation. Purely relying on VIS as a surrogate for worsening myocardial function, the need for escalating support was clouded by altering our choice of inotropes (which may generate the same effect as the prior vasoactive agent at a lower dose). Therefore, in our series, using an alternate vasoactive agent (*i.e.*, inodilator replaced by a vasoconstrictor) apparently decreased our VIS, but clinically heralded ECLS initiation.

All the patients had a normal renal function in the preoperative period (median serum creatinine value of 0.3 and IQR of 0.2 to 0.4). Eight (29.6 %) patients required PD while on ECLS (the PD catheter was inserted using closed technique with ACT between 180-200 seconds) and 16 (59.2 %) had a hemofilter in the ECMO circuit. A negative fluid balance could be achieved by diuresis and/or PD in all patients within the first 24 hours.

### Weaning from ECLS

**Fig. 2:**
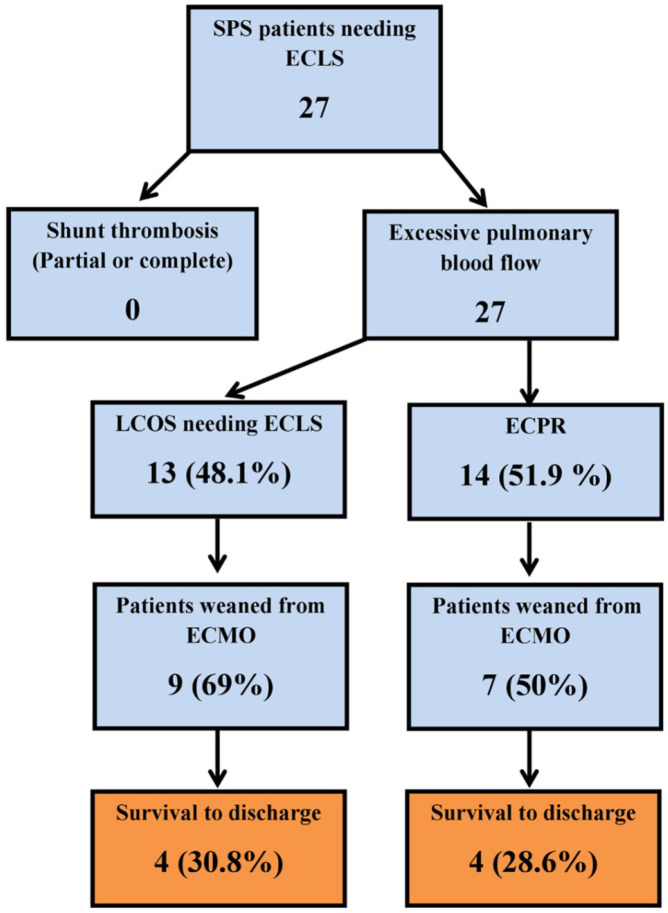
Patients’ characteristics. Out of 27 systemic to pulmonary artery shunt (SPS) patients needing extracorporeal life support (ECLS), 16 (59.3 %) patients survived ECLS weaning, and eight (29.6%) patients survived to discharge. ECMO=extracorporeal membrane oxygenation; ECPR=extracorporeal cardiopulmonary resuscitation; LCOS=low cardiac output syndrome.

Mean ECMO duration was 58.5 hours (LCOS-ECMO - 76.6 hours, ECPR - 44.6 hours). Eleven (40.7%) patients could not be weaned from ECLS (10 - myocardial failure due to distension injury, one - care withdrawal due to significant neurological damage). Sixteen (59.3%) patients (nine - LCOS-ECMO group, seven - ECPR group, *P*=0.317) were successfully weaned off ECLS. All 16 had patent, functioning SPS. Weaning strategy was ECMO bridging in three (18.8%) patients and direct weaning in 13 (81.2%) patients. Out of the 16 (59.3%) patients who survived to decannulation, eight (29.6%) patients survived to discharge (Figure 2).

**Fig. 3:**
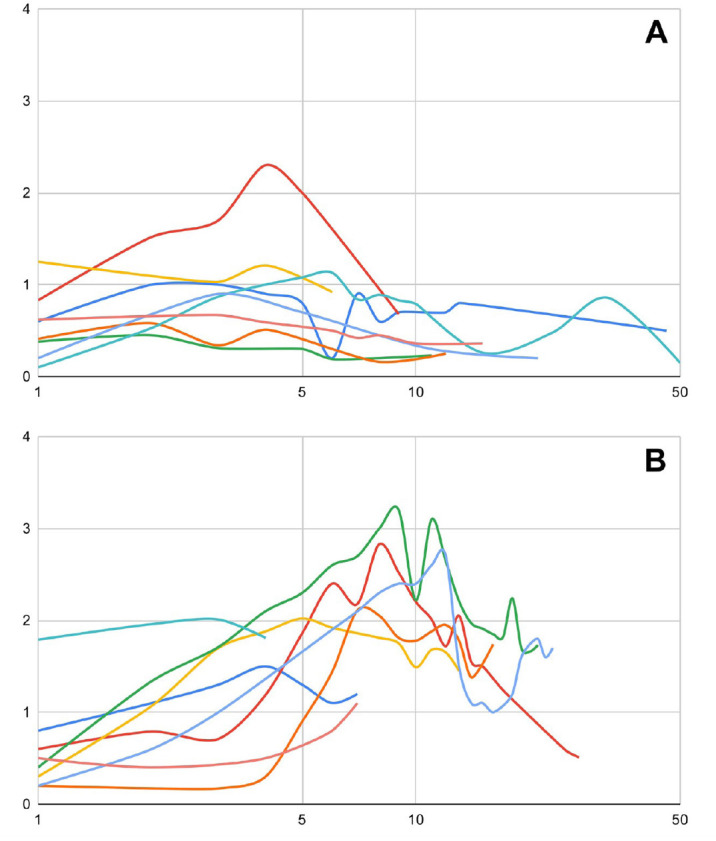
Serum creatinine trend in patients who were weaned from extracorporeal life support (ECLS). X axis shows days from surgery in logarithmic scale, Y axis shows serum creatinine in mg/dl. A) Serum creatinine trend in patients who were successfully weaned from ECLS and survived to hospital discharge. B) Serum creatinine trend in patients who were successfully weaned from ECLS but did not survive.

Average ventilation duration post ECLS was 382.4 hours. One (3.7 %) patient required tracheostomy. Additional eight patients required PD post weaning from ECLS. None of the patients had PD-related complications. Median duration for normalization of serum creatinine in patients who were successfully weaned from ECLS and survived to hospital discharge was 4.5 days (range 1-15 days). Out of the patient cohort that was successfully weaned from ECLS, but did not survive, only one patient had normalisation of serum creatinine at 25 days (Figure 3). This supports the concept that renal function is one of the best indicators of survival which is common knowledge for both cardiac and non-cardiac patients needing ECLS^[11,12]^.

### Mortality

There were 19 (70.3%) deaths in the series. Causes of death are shown in Table 1. Mortality risks were similar when ECLS was instituted for ECPR or for LCOS (*P*=0.946). There was no significant difference in mortality between the patients with pulmonary atresia (12/17, 70.6%) and the patients with antegrade flow (7/10, 70%).

**Table 1:** Patients’ cause of death.

Cause of death		Number
Could not wean from extracorporeal life support	Myocardial failure (10)	11
	Neurological issues (1)	
Bacterial sepsis	Pneumonia (1)	5
	Blood sepsis (4)	
Cytomegalovirus encephalitis (DiGeorge with absent thymus)		1
Fungal sepsis		1
Respiratory arrest (blocked tracheostomy tube)		1
Total		19

Total numbers of all-cause pediatric ECLS for cardiac indications during the same time period were 238, with successful ECMO weaning in 138 (58%) patients and 89 (37.4%) survivals to discharge. There was no statistical difference in survival to discharge between SPS-ECLS and all cause ECLS during the study period (*P*=0.428).

## DISCUSSION

SPS can be constructed as a first-stage palliation for cyanotic congenital heart disease or as part of other procedures like the Norwood operation. SPS is considered a high-risk procedure, with mortality varying between 4% to 16% depending on the age of the patient, indication for surgery, and preoperative status^[1-3]^. Mortality for a neonatal SPS is higher than for other subsets. Petrucci et al.^[3]^ reported a mortality of 7.2%, with the maximum number of deaths occurring within the first 24 hours after the operation, and nearly half of all the mortalities within 48 hours of the operation. This study showed 38% survival to hospital discharge for the patients who required ECMO support after SPS^[3]^.

**Fig. 4:**
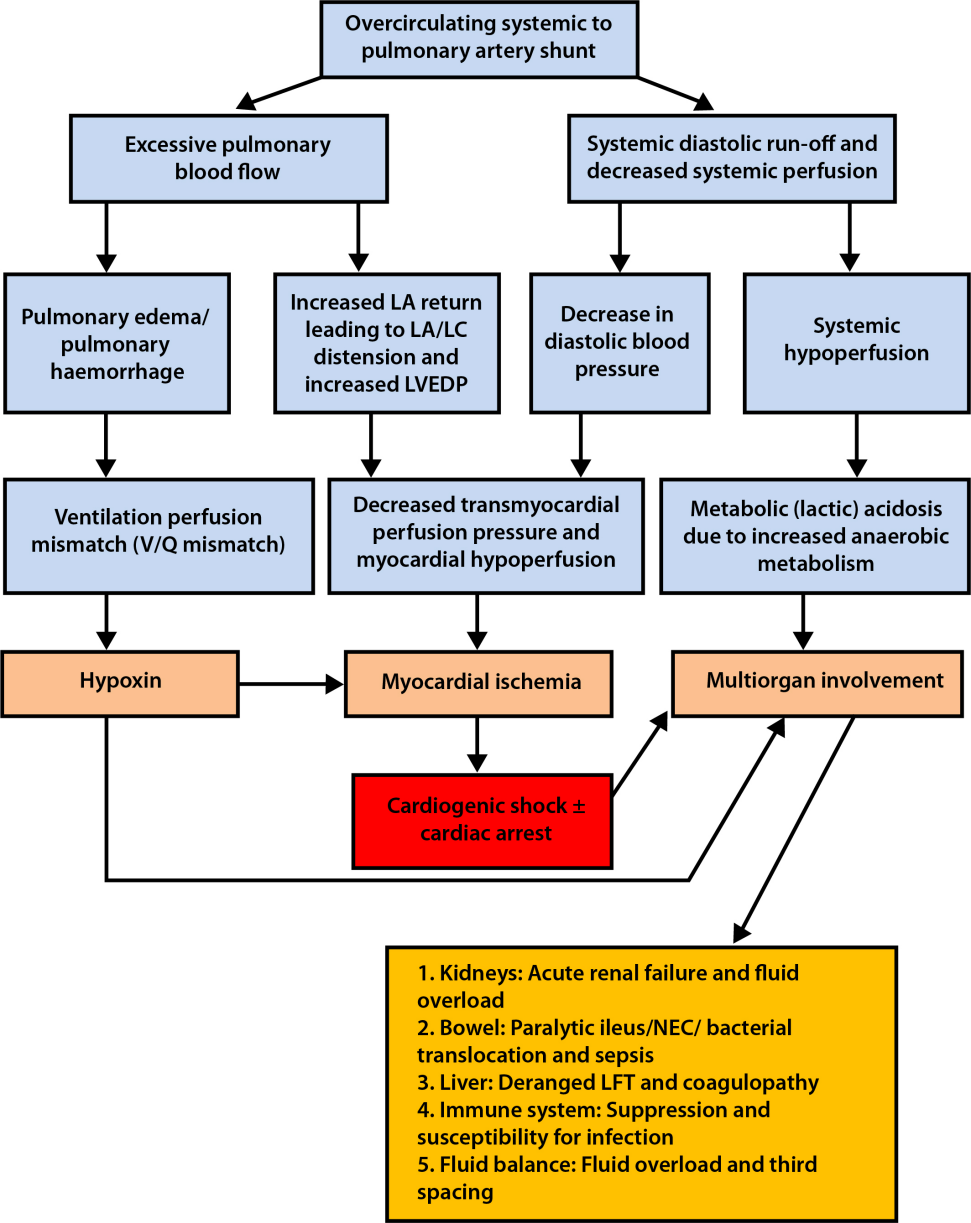
Pathophysiology of systemic to pulmonary artery shunt with excessive pulmonary blood flow. LA=left atrial; LFT=liver function test; LV=left ventricular; LVEDP=left ventricular end-diastolic pressure; NEC=necrotizing enterocolitis.

ECLS is indicated for post procedure refractory LCOS or cardiac arrest. This is usually due to excessive shunt flow but may also be due to partial or complete shunt thrombosis or because of myocardial dysfunction due to the primary procedure (in addition to shunt dynamics). The mechanism of a high flow shunt leading to refractory LCOS or cardiac arrest is depicted in Figure 4. It is known that infants who suffer major adverse events (cardiac arrest, chest reopening, or ECMO requirement) due to “overshunting” experience considerably poorer outcomes than those who experience events due to shunt blockage^[13]^. A hypoxic event with maintenance of systemic perfusion (as often seen in a blocked shunt) is probably less likely to result in poorer outcomes than those after a hypoxic-ischemic event (commonly seen in overshunting).

Complete occlusion of the shunt by means of a surgical clip at the time of initiation of ECMO was found to have unacceptable mortality, and thus many centers have adopted a strategy of increased flow to compensate for shunt runoff based on the initial publication by Jaggers et al.^[5]^. They reported outcomes in nine patients with shunts requiring ECMO. The authors occluded the shunt surgically at the time of ECMO initiation in four patients to prevent pulmonary overcirculation and had no survivors in this group. Two out of these four patients had documented pulmonary infarcts on autopsy. In contrast, in the five patients in whom the shunt was left patent and the ECMO flows were increased to compensate for the shunt runoff, mortality was considerably lower, at 20%. Most centers followed this practice, and the publication led to the strategy of leaving the shunt open to perfuse the lungs during the ECMO run, increasing flows to satisfy the needs of both the systemic and pulmonary circulation combined with normal ventilation, to let the infant balance his own systemic-pulmonary circulation. The team substantiated their findings with animal experimentation^[14]^.

Patients with SPS who suffer a major adverse event due to overshunting experience considerably poorer outcomes than those who experience events due to shunt blockage^[13]^. Most publications which claim better outcomes with an open SPS while on ECLS and running the pump at higher flow have not separated the outcomes based on ECLS for shunt thrombosis (partial or complete) *vs.* high flow-related LCOS-ECLS and ECPR. If we focus our attention on only patients with a SPS in whom ECLS was required for LCOS or cardiac arrest contributed to by excessive shunt flow, there will be a significant change in the inference.

Allan et al.^[15]^, in their study of 44 patients with shunted single-ventricle circulation supported with ECMO, followed the strategy of open SPS and increasing the ECMO flows to manage the systemic circulation. They demonstrated a 48% survival to hospital discharge. But if in the same series only the patients who needed ECMO for myocardial failure or for cardiac arrest had been considered, the survival to discharge would have been 23% (six out of 26 patients survived to hospital discharge). In the same article, the authors mention that they narrowed the shunt using one or more surgical clips, when there was inadequate systemic perfusion while on ECLS and increased flow failed to attain adequate systemic perfusion (indicating that not all patients can be managed by increasing the flows). Shunt clipping was required more frequently in patients cannulated for cardiovascular collapse. More non-survivors than survivors had their shunts clipped. However, authors hypothesized that the need for shunt clipping is a marker of severity of illness and is associated with increased mortality for that reason.

Botha et al.^[16]^ followed the strategy of increased ECLS flows without surgically restricting the shunt diameter and provided successful circulatory support in the majority of patients with SPS. Significantly higher ECMO flows were needed in the patients with SPS. A greater proportion of patients in the shunt group required > 48 hours to achieve lactate clearance and the median time to achieve lactate clearance was significantly longer in the shunt group. The median time to achieve a 12-hour period of overall negative fluid balance was longer in the shunt group. Survival for the entire SPS cohort requiring ECLS was 49%, but mortality for cardiac arrest or cardiovascular instability due to LCOS cannot be inferred from the article. And whenever ECLS was established via peripheral cannulation, the arterial cannula was placed at the junction of the innominate artery and aorta, obstructing the systemic end of the shunt, which was 19.6% of the shunt patients (equivalent to shunt restriction).

A study from Toronto which analysed functional single-ventricle palliation requiring postoperative ECLS showed a survival to hospital discharge of 44%^[17]^. Out of 25 patients in the study group, 20 had SPS and the unit followed the strategy of not restricting the shunt while on ECLS and managing the systemic circulation by increasing the ECMO flow. But even in this series when there was failure to maintain adequate systemic flow by said strategy, they had to clip shunts in two patients, and out of the eight SPS patients who were on ECLS due to a cardiac arrest, only three survived (37.5% survival to discharge).

Polimenakos et al.^[18]^ in their outcome analysis for post-cardiotomy ECPR in neonates with complex single ventricle had 57% survival to hospital discharge. But if we consider the SPS patients in the series (managed by increasing the flow while on ECLS), there were six patients and three hospital mortalities (50% survival to discharge).

Having analysed the aforementioned studies and drawing from our own experience, we can conclude that clipping/completely occluding the shunt while on ECLS will not always result in mortality. On the contrary, in situations when the indication for ECLS was a high flowing shunt, clipping has proved to be beneficial. Therefore, we feel these two subsets need to be dealt with individually, rather than in a pooled fashion, which may have skewed data in favour of keeping the shunt open.

### Limitations

1. Retrospective analysis
2. Higher proportion of sepsis-related mortality (unlike developed countries)
3. Higher mean age (due to delayed presentation)
4. Absence of Norwood patients
5. We did not have serum lactate measuring facilities during the initial half of the study period; hence lactate trends could not be reported in the data in any useful way.

## CONCLUSION

Increased flow to maintain systemic circulation for a SPS patient while on ECLS is an accepted strategy, but it should probably not be applied universally. We acknowledge that conclusions are difficult to establish, however a large subset of SPS patients, who require ECLS either due to a cardiac arrest or refractory LCOS due to a high shunt flow, might benefit from a strategy of completely occluding the shunt soon after commencement of ECLS, especially when there is frank pulmonary edema or haemorrhage in the pre-ECLS period. In such patients, restricting the shunt soon after establishment of ECLS might provide better systemic perfusion, faster clearance of pulmonary edema or haemorrhage, early clearance of metabolic acidosis, and avoid positive fluid balance immediately after ECLS establishment. And on the contrary, establishment of ECLS in the presence of frank pulmonary edema/haemorrhage with a strategy of increased flow might be actually detrimental. We suggest that a prospective randomised trial is ethically justified for the subset of patients receiving ECLS for the indication of excessive pulmonary blood flow.
